# Successful Treatment of Paraneoplastic Neuropathy and Pruritis With Scrambler Therapy: A Case Report

**DOI:** 10.7759/cureus.26861

**Published:** 2022-07-14

**Authors:** Tyler K Murphy, C A Pardo, Ricardo H Roda, Rebecca L Stone, Thomas J Smith

**Affiliations:** 1 Medicine, Johns Hopkins University School of Medicine, Baltimore, USA; 2 Neurology, Johns Hopkins University School of Medicine, Baltimore, USA; 3 Gynecologic Oncology, Johns Hopkins University School of Medicine, Baltimore, USA; 4 Oncology, Hospice and Palliative Medicine, Sidney Kimmel Comprehensive Cancer Center, Johns Hopkins Medicine, Baltimore, USA

**Keywords:** nodular hodgkins, scrambler therapy, neuromodulation, pruritis, paraneoplastic neurologic syndrome

## Abstract

Paraneoplastic neuropathy, including pruritis, remains a vexing problem as it often does not resolve even with successful treatment of cancer. Scrambler Therapy is a superficial form of neuromodulation that replaces the pain signal with “non-pain information” that is approved for chronic and neuropathic pain, with few side effects. We report here two cases of paraneoplastic neuropathy, one with additional pruritis, that both responded satisfactorily to Scrambler Therapy with no side effects.

## Introduction

Paraneoplastic syndromes are rare, affecting one in 10,000 cancer patients, and paraneoplastic neuropathy is one of the most common syndromes. These paraneoplastic conditions, while associated with an underlying malignancy, are not the result of tumor invasion, metastasis, or the consequence of any treatments [1]. Sensory neuropathy (SN) is often severe and refractory to usual treatments and persists after the cancer is cured. The most common malignancies responsible for paraneoplastic neurological syndromes are small cell lung cancer, and breast and gynecological malignancies, although other neoplasms may be responsible as well [2,3]. Many patients often suffer from neurologic symptoms before diagnosis of their underlying malignancy, sometimes by a matter of years. Damage done to multimodal C-fibers by the immune system is thought to be the etiology of the neurologic symptoms. Despite the underlying autoimmune process, approximately 30%-40% of paraneoplastic neurologic syndromes can occur without antibodies [1,4]. Multimodal C-fibers are also responsible for itch sensations, leading some patients with paraneoplastic polyneuropathy to experience pruritis as well [5,6]. Treatment of paraneoplastic neurologic disorders relies on treatment of underlying tumor when possible, immune suppression, as well as symptom control, frequently accomplished through pharmacologic means [7]. Tumor ablation is sometimes effective, when possible, at preventing symptom progression. For patients with paraneoplastic peripheral neuropathy syndromes associated with cell surface antibodies, immune suppression therapies such as plasma exchange, intravenous immunoglobulin, and steroids have provided some benefits [8]. Pharmacologic treatments for paraneoplastic pain and pruritis are available, including tricyclic antidepressants (TCAs), serotonin noradrenaline reuptake inhibitors (SNRIs), and anticonvulsants such as gabapentin and pregabalin, but all are known to cause significant side effects in some patients [9].

Scrambler Therapy has been shown to provide benefits for neuropathic pain through neuromodulation [10]. The theory behind Scrambler Therapy’s mechanism of action is that by replacing endogenous pain signals with synthetic signals, pain can be improved or resolved. Using topical EKG electrode pairs, these synthetic signals can be transmitted via the surface receptors of C-fibers using topical electrical stimulation channels [11-13]. These treatments are repeated over multiple days, which serves to “retrain” the brain leading to improvement in neuropathic symptoms. Overall, Scrambler Therapy represents an inexpensive and minimally invasive treatment option for patients experiencing some types of neuropathic pain, and perhaps pruritis [14].

We report here a case of refractory paraneoplastic SN and pruritis, and one of paraneoplastic neuropathy treated successfully with Scrambler Therapy.

## Case presentation

Case 1

This 66-year-old man presented for evaluation and treatment of peripheral neuropathy and pruritis. Three years prior, he suddenly developed a sensation of “heat” in his left foot, which he likened to “walking on hot sand.” Over the course of two weeks, this sensation spread to his right foot, then began to spread up the calves to mid-shin bilaterally. Coincident with the neuropathy, he developed severe pruritis on his back, as well as both flanks, unresponsive to topical agents including steroids and anti-histamines. The patient subsequently underwent treatment trials with gabapentin, duloxetine, topical menthol, and pregabalin, none of which helped either of his symptoms. Neuropathic symptoms progressed to the point that patient noted an altered gait due to pain and difficulty feeling his toes and the balls of his feet. About six months later, he began to experience weight loss, weakness, and fatigue. He continued to have the burning neuropathy plus pins and needles sensation in his feet. Imaging showed cervical spinal stenosis, and the patient underwent a C2-C7 laminectomy and laminoplasty in 2021. Over the following four months, the patient continued to lose weight, up to 30 pounds, and further investigation with a PET scan showed multiple enlarged lymph nodes in the right groin. A biopsy was performed with results showing classical Hodgkin’s Lymphoma. Because of the neuropathy, he was treated with three cycles of cyclophosphamide, hydroxyldaunorubicin, etoposide, and prednisone; etoposide was substituted to avoid neurotoxicity with vincristine [15]. He does not remember if the prednisone helped his pruritis. He subsequently had involved field external beam radiation and has remained in remission for the past year.

He presented to Johns Hopkins Hospital at age 66 for rehabilitation. Initial examination showed absent deep tendon reflexes in the legs but was otherwise normal, and there were no abnormal skin findings. A review of prior EMGs showed axonal degeneration. The Mayo Clinic Panel for paraneoplastic disorders was negative. He remained on gabapentin 900 mg in the morning and 1,800 mg at night, mostly to help him sleep.

**Figure 1:**
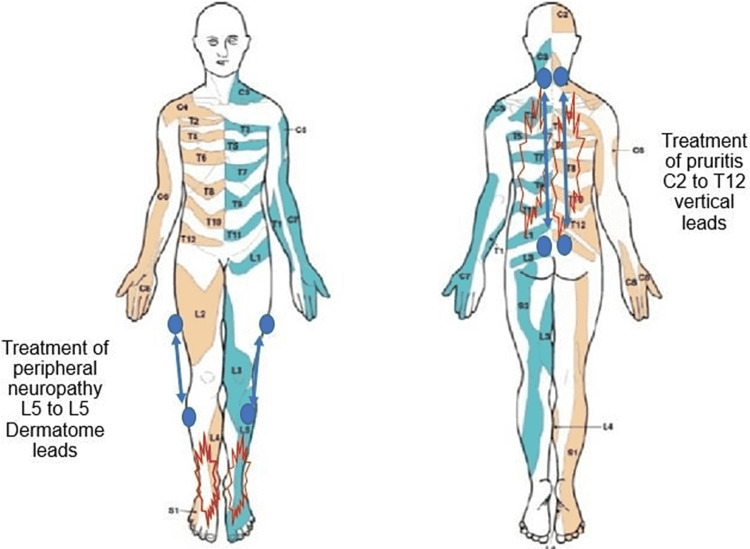
Location of patient’s reported neuropathy and pruritis, and respective electrode placements

He was treated with five sessions of Scrambler Therapy, on consecutive days. His foot pain diminished from 8/10 to at least 3/10, and he regained normal motion of his toes and forefoot. His gait improved such that he could jog for six minutes. The tingling in his feet was similarly improved. The itching on his back was “substantially reduced” but still present. Both reliefs lasted about four weeks before returning. The electrode setup for treating peripheral neuropathy and pruritis is shown in Figure 1.

**Figure 2:**
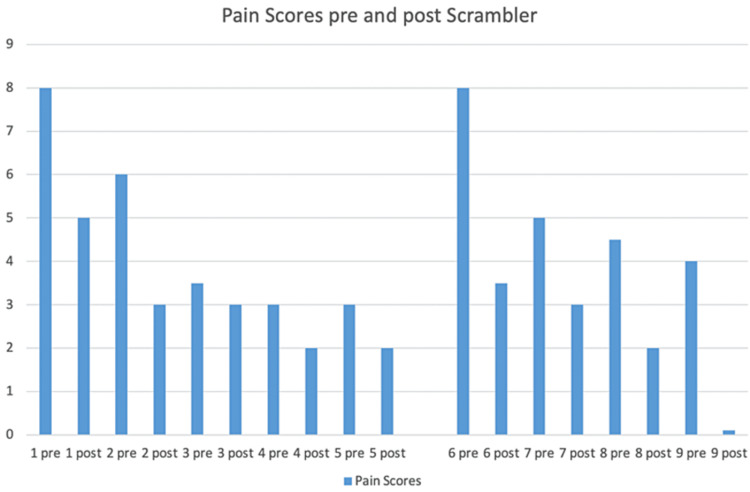
Pain response to Scrambler Therapy (“0” shown as 0.1 for illustration)

He returned for four more consecutive treatment sessions approximately six weeks after the initial five consecutive treatments. The patient reported 8/10 pain on his initial follow-up appointment, but over the course of four consecutive follow-up treatments, was able to achieve significant pain reduction to zero and almost complete resolution of the pruritis. Two months later, his pain and pruritis remain well controlled and do not require treatment (Figure 2).

Case 2

This 52-year-old woman presented two weeks prior to Thanksgiving 2020 with difficulty going up and down stairs, and generalized leg weakness. Previously, she had been a runner and taught cycling classes. This did not respond to steroids. An EMG was normal, as were all lab tests. A CT scan done to evaluate lumbar neuropathy disclosed adenopathy adjacent to the right common iliac vasculature and anterolateral to the sacrum on the right. A repeat EMG was normal, showing no sensory loss. A pelvic lymph node biopsy done in March 2021 showed squamous cell carcinoma with lymphoepithelial features, HPV positive. PET scan showed a mass in the cul de sac of Douglas and bilateral iliac lymphadenopathy, consistent with cervical primary cancer. She was diagnosed with a FIGO stage IIIC2 squamous cell carcinoma of the cervix and underwent concurrent weekly cisplatin and external beam radiation to the whole pelvis followed by brachytherapy ending June 2021. In May 2022, she was referred for Scrambler Therapy and complained of deep aching pain with some burning dysesthesias in both legs, from the proximal thighs to the ankles. Her pain scores ranged from 5 to 8/10. Hands and feet were spared. She underwent five days of Scrambler Therapy, with the electrodes placed above the pain in L5, L4, and S1 dermatomes, with the distal electrodes placed on the feet below the pain. Her allodynia and hypersensitivity diminished within 10 minutes and by the end of day 5, she was essentially pain-free. Without the pain, she was able to walk normally again for the first time in 18 months. One month later, she is mostly without pain, only having some muscle pain after exercise.

Both patients gave written permission to report their cases (available on demand). Johns Hopkins IRB does not require approval for case reports of three or fewer subjects.

## Discussion

Scrambler Therapy is a non-invasive method of neuromodulation, using EKG electrode pairs to capture the terminal ends of c-fibers and send “non-pain information” to the brain, in theory reducing central sensitization. To our knowledge, it has not been used before in the treatment of paraneoplastic neuropathy or pruritis, which often does not clear after cancer treatment, and is often not well controlled with medication. the diagnosis of paraneoplastic neuropathy is still on clinical criteria. Electromyography (EMG) is rarely helpful in determining the etiology or documentation of paraneoplastic peripheral neuropathy; in one recent study, only two of 32 (6%) patients had abnormal EMG findings [16]. We could find no other cases in the medical literature treated with Scrambler Therapy. This was accomplished with no medication side effects and appears to be long-lasting. In addition, people who respond once will always respond again, in our experience.

## Conclusions

This case adds to a growing list of neuropathic problems successfully treated with Scrambler Therapy, from central pain after a stroke to diabetic peripheral neuropathy. The Food and Drug Administration approved it for both chronic and neuropathic pain, without specifying types. The complete absence of side effects makes this very appealing. In addition, a one-time expenditure for Scrambler Therapy may cost much less than a lifetime of expensive neuropathic pain drugs and makes the use of opioids unnecessary. Given the good response to Scrambler Therapy presented in these cases and the absence of any side effects, we believe that further research is indicated to further evaluate the most appropriate role of this modality in the treatment of paraneoplastic neuropathy and that patients should be aware of this potentially very helpful modality.
